# RWCFusion: identifying phenotype-specific cancer driver gene fusions based on fusion pair random walk scoring method

**DOI:** 10.18632/oncotarget.11064

**Published:** 2016-08-05

**Authors:** Jianmei Zhao, Xuecang Li, Qianlan Yao, Meng Li, Jian Zhang, Bo Ai, Wei Liu, Qiuyu Wang, Chenchen Feng, Yuejuan Liu, Xuefeng Bai, Chao Song, Shang Li, Enmin Li, Liyan Xu, Chunquan Li

**Affiliations:** ^1^ School of Medical Informatics, Daqing Campus, Harbin Medical University, Daqing, 163319, China; ^2^ The Key Laboratory of Molecular Biology for High Cancer Incidence Coastal Chaoshan Area, Shantou University Medical College, Shantou, 515041, China; ^3^ School of Life Sciences and Biotechnology, Shanghai Jiao Tong University, Shanghai, 200240, China; ^4^ Department of Mathematics, Heilongjiang Institute of Technology, Harbin, 150050, China; ^5^ School of Nursing and Pharmacology, Daqing Campus, Harbin Medical University, Daqing, 163319, China; ^6^ College of Bioinformatics Science and Technology, Harbin Medical University, Harbin, 150081, China

**Keywords:** gene fusion, cancer, network, driver

## Abstract

While gene fusions have been increasingly detected by next-generation sequencing (NGS) technologies based methods in human cancers, these methods have limitations in identifying driver fusions. In addition, the existing methods to identify driver gene fusions ignored the specificity among different cancers or only considered their local rather than global topology features in networks. Here, we proposed a novel network-based method, called RWCFusion, to identify phenotype-specific cancer driver gene fusions. To evaluate its performance, we used leave-one-out cross-validation in 35 cancers and achieved a high AUC value 0.925 for overall cancers and an average 0.929 for signal cancer. Furthermore, we classified 35 cancers into two classes: haematological and solid, of which the haematological got a highly AUC which is up to 0.968. Finally, we applied RWCFusion to breast cancer and found that top 13 gene fusions, such as BCAS3-BCAS4, NOTCH-NUP214, MED13-BCAS3 and CARM-SMARCA4, have been previously proved to be drivers for breast cancer. Additionally, 8 among the top 10 of the remaining candidate gene fusions, such as SULF2-ZNF217, MED1-ACSF2, and ACACA-STAC2, were inferred to be potential driver gene fusions of breast cancer by us.

## INTRODUCTION

Human genomic aberrations have been proved to be causal factors of many diseases. Among the most widely studied variations, gene fusions have been of great interest due to their important role in human cancer. It has been estimated that 20% of human cancer are caused by gene fusions [1]. There are strong evidence showing that gene fusions drive the initial step and the development of cancer, and thus are potential prognostic tools or therapeutic targets in anti-cancer treatment. A convincing example is fusion gene BCR-ABL, which can be translated to an abnormal fusion protein-tyrosine kinase and drive the development of chronic myelogenous leukemia (CML). And the drug Glivec which target BCR- ABL chimeric protein has been has been proved very successful in the treatment of CML [2, 3]. Nowadays, several fundamental databases can provide fusion events involved in cancer. To our best knowledge, the following databases TICdb [4], dbCrid [5], ChimerDB 2.0 [6], Mitelman [1], and ChiTaRS [7] contain fusion events of human cancers.

The advances in next-generation sequencing (NGS) technologies help to detect gene fusions. Several tools based on next-generation sequencing such as Tophat-Fusion [8] and deFuse [9] are able to effectively identify novel fusion transcripts through aligning pair-end RNA-seq reads. These methods accelerated the discovery of tens to hundreds of gene fusions in various cancers, including solid tumors and hematological disorders [10]. However, recent studies demonstrated that minority of the gene fusions detected by next-generation based tools are important driver fusions for cancer development and most were just nonspecific passengers [1]. In a word, these next-generation sequencing based methods help to detect gene fusions in cancer tissues, but they failed to identify driver gene fusions.

Thus, developing a method to identify driver gene fusions was urgent. Until now, only a few attempts have been done to do this. One well established approach named Consig to distinguish driver from passenger gene fusions was proposed by Wang et al. It nominates driver fusions by the direct association of partner genes with identified fusion concept signatures, generated through enrichment of established fusions from the Mitelman database against all concepts compiled from molecular interactions, functional annotations and pathways [11]. This method ignored the specificity of phenotype when deducing fusion concept signatures. In addition, it is a single gene-based approach, only accounting for the impact of a single gene fusion. Protein-protein interaction network is a valuable resource for prioritization candidate gene fusions, because genes related to a specific or similar disease phenotype tend to be located in a specific neighborhood [12]. A computational approach named fusion centrality toward prioritization of candidate gene fusions that study network properties have been developed by Wu et al. The authors hypothesized that the partner genes of the driver fusions were prone to present as hubs in a network [13]. Although providing a useful tool to identify driver gene fusions, this approach also failed to account for the specificity among different cancers when using protein-protein interaction network. This means that for different cancers, a certain gene fusion candidate would get a same score by fusion centrality. What's more, because it only considered the impact of direct neighbors, it only explain the local topological property of network, ignoring global features.

In this work, we proposed a phenotype specific computational method called RWCFusion based gene interaction network to identify the driver gene fusions. First, we constructed a weighted gene interaction network from protein-protein interaction (PPI) network in STRING database [14] and mapped the high-risk cancer gene fusions into the network. Then, we developed a novel fusion pair random walk scoring method in the global network to identify phenotype-specific driver gene fusions with high-risk fusion genes acting as seed nodes. This strategy could exploit the global topology information of the network and identify phenotype-specific driver gene fusions according to the similarity between candidate fusions and seeds in the network. We evaluated the performance of RWCFusion on 483 candidate gene fusions corresponding to 35 cancer phenotypes by leave-one-out cross-validation and it achieved a high overall AUC value of 0.925 and an average of 0.929, which was much higher than other existing methods. Furthermore, we divided the 35 cancers into haematological and solid classes. Results showed they both had a good performance, especially the haematological, which got an AUC value of 0.968. Finally, we applied RWCFusion to breast cancer data to identify driver fusion genes. There were 13 fusion pairs in the top having been proved to be high-risk for breast cancer, for example, BCAS3-BCAS4, NOTCH-NUP214, MED13-BCAS3 and CARM-SMARCA4. Additionally, 8 among the top 10 of the remaining candidate gene fusions, such as SULF2-ZNF217, MED1-ACSF2, and ACACA-STAC2, were inferred to be potential driver gene fusions of breast cancer.

## RESULTS

### Performance of the RWCFusion

To evaluate and execute RWCFusion, we need a basic gene interaction network and seed gene fusions at first. The gene interaction network we constructed in this article contains 16785 genes and 1515370 edges. And the seed gene fusions contains 483 high risk gene fusions altogether, corresponding to 35 phenotypes.

To evaluate the performance of RWCFusion, we used leave-one-out cross-validation for every high-risk gene fusion and plotted ROC for each of the 35 cancers, haematological, solid cancer and overall cancers separately. The ratio of all high risk gene fusions (positive) to total virtual background fusion pairs (virtually negative) is 3.4e-06 while the ratio of all involved high risk partners to all genes in the network is 3.0 e-02. Taking this into account, we balanced the test set with 1 positive gene fusion and 99 negative gene fusion in each test case of leave-one-out cross-validation [15].

**Figure 1:**
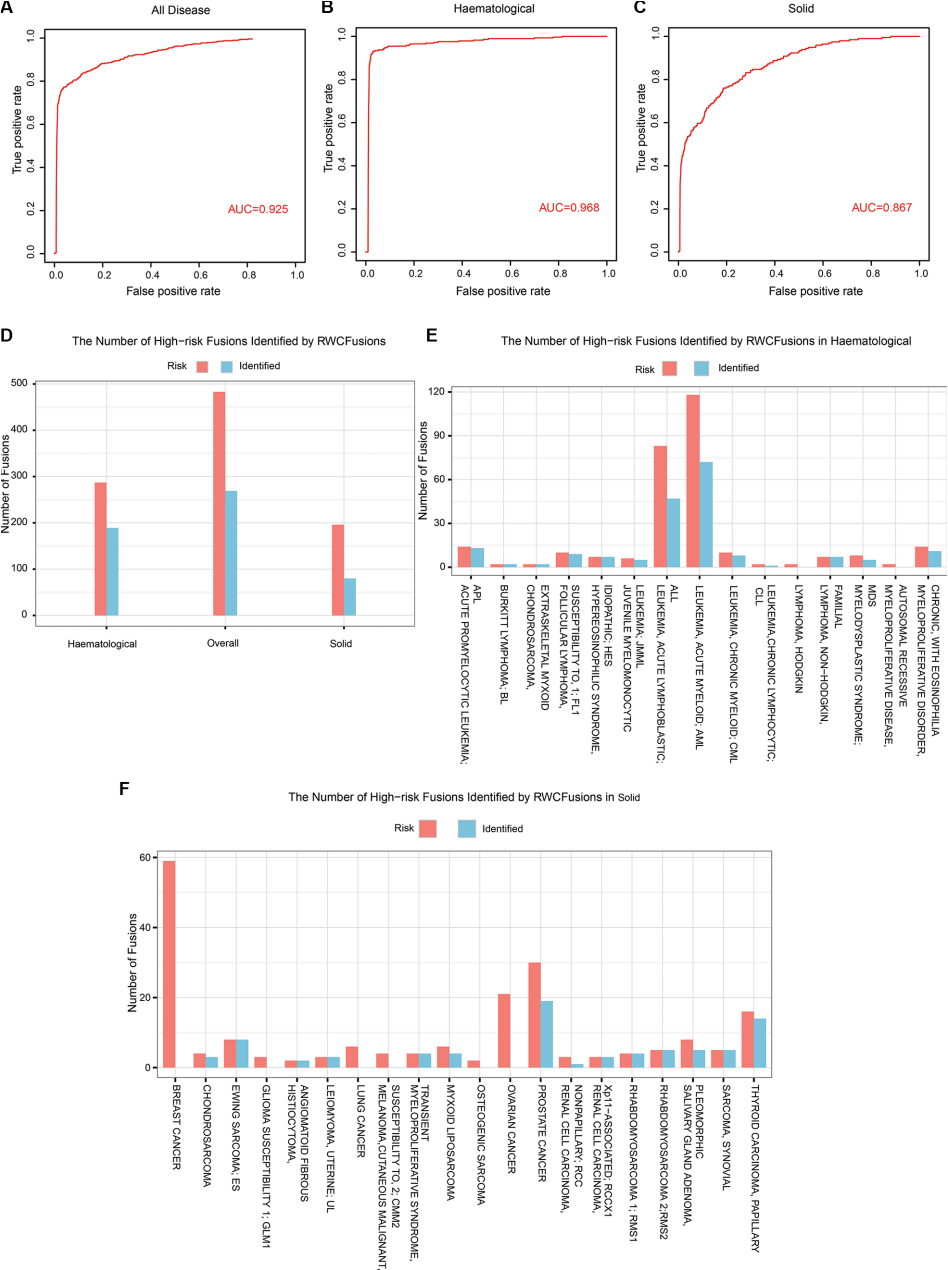
The performance of RWCFusion evaluated by leave-one-out cross validation (**A**, **B** and **C**) showed the AUC results for overall cancers, haematological and solid classes respectively based on leave-one-out cross validation. (**D**, **E** and **F**) showed the number of priviously known high-risk gene fusions identified by RWCFusion.

Using RWCFusion, we got a high overall AUC value of 0.925 (Figure 1A), and a better one of 0.968 for haematological cancer (Figure 1B). For the other class solid, the AUC value is 0.867 (Figure 1C). And for 35 single cancer phenotype, 28 of them, such as haematological cancer CHRONIC MYELOID, MYELODYSPLASTIC SYNDROME and solid cancer THYROID CARCINOMA, LEIOMYOMA, got an AUC value higher than or equal to 0.913 (Table 1). The mean AUC value for 35 cancers is 0.929. As is shown, haematological cancer got a high AUC value than solid cancer. We all know that genes in blood are easier to be detected than in solid tissues, which might indicate that Tophat-Fusion would got a better accuracy with haematological cancer RNA-seq data than solid when obtaining candidate gene fusions. And this might affect the performance of RWCfusion to identify drivers in candidate gene fusions in cancers. Altogether, the AUC results showed that RWCFusion performed all well for overall cancers, haematological and solid caner classes, and single cancer.

**Table 1:** AUC performance of RWCFusion and existed method fusion centrality for single 35 single, haematological, solid and overall phenotype

OMIM ID	Disease name/class	AUC value		
		RWCFusion	Centrality	RWCFusion-Centrality
159595	MYELOPROLIFERATIVE SYNDROME, TRANSIENT	0.997	0.970	0.027
613024	FOLLICULAR LYMPHOMA, SUSCEPTIBILITY TO, 1; FL1	0.997	0.969	0.028
613065	LEUKEMIA, ACUTE LYMPHOBLASTIC; ALL	0.949	0.873	0.076
113970	BURKITT LYMPHOMA; BL	0.997	0.997	0
114480	BREAST CANCER	0.784	0.700	0.084
131440	MYELOPROLIFERATIVE DISORDER, CHRONIC, WITH EOSINOPHILIA	0.997	0.975	0.022
137800	GLIOMA SUSCEPTIBILITY 1; GLM1	0.759	0.763	−0.004
144700	RENAL CELL CARCINOMA, NONPAPILLARY; RCC	0.978	0.746	0.232
150699	LEIOMYOMA, UTERINE; UL	0.996	0.690	0.306
151400	LEUKEMIA, CHRONIC LYMPHOCYTIC; CLL	0.995	0.992	0.003
155601	MELANOMA, CUTANEOUS MALIGNANT, SUSCEPTIBILITY TO, 2; CMM2	0.703	0.883	−0.18
167000	OVARIAN CANCER	0.624	0.598	0.026
176807	PROSTATE CANCER	0.914	0.794	0.12
181030	SALIVARY GLAND ADENOMA, PLEOMORPHIC	0.993	0.838	0.155
188550	THYROID CARCINOMA, PAPILLARY	0.995	0.954	0.041
211980	LUNG CANCER	0.914	0.843	0.071
215300	CHONDROSARCOMA	0.964	0.781	0.183
236000	LYMPHOMA, HODGKIN	0.657	0.667	−0.01
259500	OSTEOGENIC SARCOMA	0.73	0.707	0.023
268210	RHABDOMYOSARCOMA 1; RMS1	0.997	0.959	0.038
268220	RHABDOMYOSARCOMA 2;RMS2	1	0.968	0.032
300854	RENAL CELL CARCINOMA, Xp11-ASSOCIATED; RCCX1	1	0.731	0.269
601626	LEUKEMIA, ACUTE MYELOID; AML	0.98	0.903	0.077
605027	LYMPHOMA, NON-HODGKIN, FAMILIAL	0.999	0.923	0.076
607685	HYPEREOSINOPHILIC SYNDROME, IDIOPATHIC; HES	0.999	0.971	0.028
607785	JUVENILE MYELOMONOCYTIC LEUKEMIA; JMML	0.994	0.981	0.013
608232	LEUKEMIA, CHRONIC MYELOID; CML	0.971	0.933	0.038
612160	HISTIOCYTOMA, ANGIOMATOID FIBROUS	1	0.995	0.005
612219	EWING SARCOMA; ES	1	0.900	0.1
612237	CHONDROSARCOMA, EXTRASKELETAL MYXOID	1	0.768	0.232
612376	ACUTE PROMYELOCYTIC LEUKEMIA; APL	0.996	0.954	0.042
613488	MYXOID LIPOSARCOMA	0.936	0.858	0.078
614286	MYELODYSPLASTIC SYNDROME; MDS	0.979	0.893	0.086
254700	MYELOPROLIFERATIVE DISEASE, AUTOSOMAL RECESSIVE	0.735	0.929	−0.194
300813	SARCOMA, SYNOVIAL	0.996	0.427	0.569
	Solid	0.968	0.903	0.065
	Haematological	0.867	0.77	0.097
	Overall	0.925	0.845	0.080

After plotting ROC curve for overall cancers, we selected a best cutoff according to positive likelihood ratio (PLR), that is, the ratio of true positive ratio to false positive ratio. To get the best cutoff, the maximum PLR was calculated. And the cutoff correspond to the maximum PLR is taken as the best cutoff [16]. When using this best cutoff (0.0002125) to the test gene fusion set of 35 cancers, we identified 269(over 50%) out of the 483 previously known high-risk cancer gene fusions Figure 1D, Supplementary Table S1, including 189 out of 287 for haematological Figure 1D and 1E, Supplementary Table S1 such as HMGA2-COX6C, HMGA2-CCNB1IP1 of uterine cancer and 80 out of 196 for solid Figure 1D and 1F, Supplementaray Table S1 such as NUP98-TOP1, NSD1-NUP98 of MDS. This indicated that RWCFusion scores could be applied to classify a certain gene fusion into risk or non-risk gene fusion of disease with appropriate cutoff.

To further explore the reliability of RWCFusion, we examined the distribution of scores in all test gene fusions in leave-one-out cross-validation, including 483 high-risk gene fusions of cancers and 47817 (483 × 99) virtual gene fusions obtained from gene interaction network. After executing leave-one-out cross-validation for all cancers, we found 81% of the 483 high-risk gene fusions ranked in the top 10% of all test gene fusions. We also analyzed the top 20% of the test set and found 425 out of the 483 high-risk gene fusions were located in the top 20%, and the ratio is 88%. What's more, 91% high-risk gene fusions ranked in the top 30% while 96% ranked in the top 50%. From this we could see that most high-risk cancer gene fusions would get high scores with RWCFusion and ranked top in the test set.

### Investigating the robustness of RWCFusion

We investigated the robustness of RWCFusion from two aspects: removing edges in the gene interaction network; setting different value of the restart probability β. For overall cancers, we calculated the AUC value after removing 10% to 50% edges of the gene interaction network separately. The result showed that RWCFusion had strong resistance against the incompleteness of the network: the AUC value only declined 0.008 when removing 10% to 50% edges (Supplementary Table S2).

To investigate the influence of restart probability *β* value, we set it to 0.1, 0.3, 0.5, 0.7 and 0.9 respectively and then calculated the AUC value for overall cancers. The result showed that the overall performance of RWCFusion were stable under the perturbation of *β* and it made no significant difference no matter what we set it to (Supplementary Table S3). And in this work, we set it to 0.7 (Supplementary Table S3).

To sum up, RWCFusion had robustness against the resistance incompleteness of the network and the restart probability *β*.

### Performance comparison of RWCFusion with existing method fusion centrality

**Figure 2:**
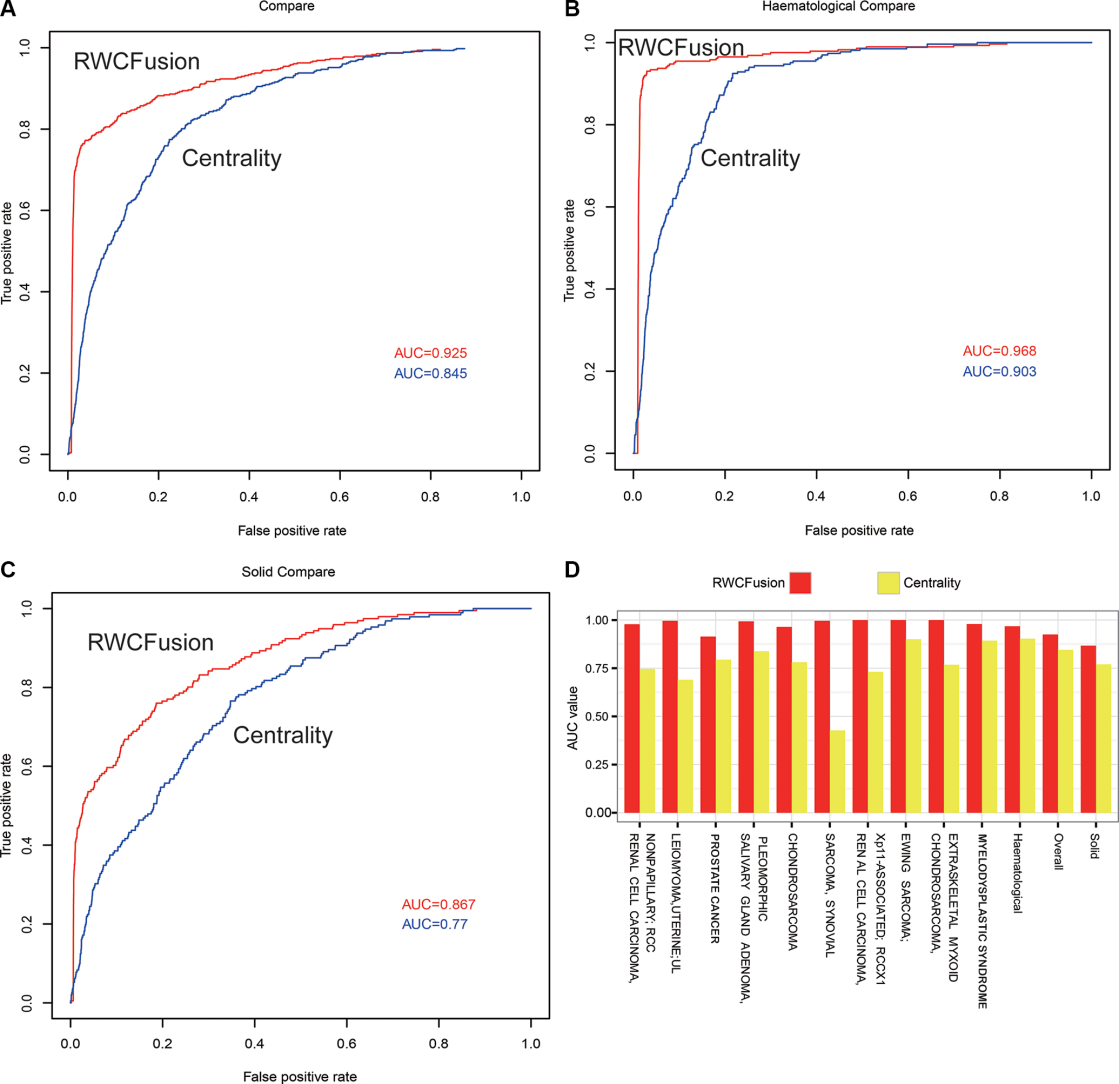
Performance compare between RWCFusion and existing method: fusion centrality (**A–C**) showed the AUC compare for overall cancers, haematological and solid classes respectively. (**D**) showed the top 10 AUC gap of single cancer.

To compare the performance of two methods, we used leave-one-out cross-validation to the 483 high-risk gene fusions corresponding to 35 cancers and calculated the AUC value for overall, two class cancers and 35 single cancers. We found the performance of RWCFusion was better than fusion centrality in overall cancers, two cancer classes and most single cancers. The overall AUC value of RWCFusion was 0.925, higher than that of fusion centrality method: 0.845 Figure 2A. And the AUC of haematological cancers was 0.968 for RWCFusion, higher than 0.903 for fusion centrality Figure 2B. As to solid cancers, it was 0.867 for RWCFusion, higher than 0.770 for fusion centrality (Figure 2C). Results also showed that RWCFusion got higher AUC value than fusion centrality in 30 out of 35 cancers Table 1, Figure 2D. One cancer got equal AUC value between the two methods and only four cancers got AUC value lower for RWCFusion than fusion centrality (Table 1).

RWCFusion is phenotype-specific compared to fusion centrality in distinguishing driver and passenger gene fusions because high-risk gene fusions, acting as seed nodes, of each phenotype are different. However, fusion centrality fail to be phenotype-specific, which will lead to a common candidate gene fusion of different cancers getting a same score, ignoring the difference between cancers. When using leave-one-out cross-validation to the 483 high-risk gene fusions corresponding to 35 cancers, we found 16 gene fusions were shared by two different cancers. The scores of them among shared cancers in fusion centrality were the same but varied in RWCFusion (Table 2). This result showed that RWCFusion is phenotype-specific, namely if a candidate gene fusion is detected in several different cancers at the same time, the RWCFusion scores of it for different cancers are also different because high-risk disease gene fusions of different cancers are different. This means RWCFusion can distinguish the importance of a certain gene fusion in different cancers.

**Table 2:** The comparison between RWCFusion and existed method fusion centrality when scoring a common candidate gene fusion which is fused in several different cancers simultaneously

gene1	gene2	OMIMID	Disease name	Cen-score	Our score
FGFR1OP	FGFR1	159595	MYELOPROLIFERATIVE SYNDROME, TRANSIENT	0.028	0.0019
		601626	LEUKEMIA, ACUTE MYELOID; AML	0.028	0.00017
FGFR1OP2	FGFR1	601626	LEUKEMIA, ACUTE MYELOID; AML	0.024	0.00012
		159595	MYELOPROLIFERATIVE SYNDROME, TRANSIENT	0.024	0.00098
NUP98	TOP1	601626	LEUKEMIA, ACUTE MYELOID; AML	0.027	0.00046
		159595	MYELOPROLIFERATIVE SYNDROME, TRANSIENT	0.027	0.00028
PAPOLA	AK7	114480	BREAST CANCER	0.0087	0.000010
		176807	PROSTATE CANCER	0.0087	0.000019
MYO9B	FCHO1	176807	PROSTATE CANCER	0.0042	0.000003
		114480	BREAST CANCER	0.0042	0.000016
PAX3	NCOA1	268210	RHABDOMYOSARCOMA 1; RMS1	0.022	0.00044
		268220	RHABDOMYOSARCOMA 2; RMS2	0.022	0.000027
MLL	CREBBP	601626	LEUKEMIA, ACUTE MYELOID; AML	0.036	0.00027
		114480	BREAST CANCER	0.036	0.00010
PAX3	FOXO1	268210	RHABDOMYOSARCOMA 1; RMS1	0.031	0.00050
		268220	RHABDOMYOSARCOMA 2; RMS2	0.031	0.00069
BCR	ABL1	601626	LEUKEMIA, ACUTE MYELOID; AML	0.040	0.00037
		608232	LEUKEMIA, CHRONIC MYELOID; CML	0.040	0.00128
BCR	JAK2	131440	MYELOPROLIFERATIVE DISORDER, CHRONIC, WITH EOSINOPHILIA	0.049	0.00057
		601626	LEUKEMIA, ACUTE MYELOID; AML	0.049	0.00033
CBFB	MYH11	601626	LEUKEMIA, ACUTE MYELOID; AML	0.015	0.00024
		608232	LEUKEMIA, CHRONIC MYELOID; CML	0.015	0.00025
FIP1L1	PDGFRA	607685	HYPEREOSINOPHILIC SYNDROME, IDIOPATHIC; HES	0.021	0.00072
		601626	LEUKEMIA, ACUTE MYELOID; AML	0.021	0.00014
CCDC6	PDGFRB	607785	JUVENILE MYELOMONOCYTIC LEUKEMIA; JMML	0.025	0.00044
		608232	LEUKEMIA, CHRONIC MYELOID; CML	0.025	0.00020
NDE1	PDGFRB	608232	LEUKEMIA, CHRONIC MYELOID; CML	0.030	0.00021
		607785	JUVENILE MYELOMONOCYTIC LEUKEMIA; JMML	0.030	0.00042
PAX7	FOXO1	268210	RHABDOMYOSARCOMA 1; RMS1	0.026	0.00042
FOXO1	PAX7	268220	RHABDOMYOSARCOMA 2; RMS2	0.026	0.00064
PDGFRB	NIN	607685	HYPEREOSINOPHILIC SYNDROME, IDIOPATHIC; HES	0.025	0.00034
NIN	PDGFRB	131440	MYELOPROLIFERATIVE DISORDER, CHRONIC, WITH EOSINOPHILIA	0.025	0.00075

Cen-score are scores of gene fusions by scoring method fusion centrality.

Our score are scores of gene fusions by our method RWCFusion.

### Case study: identify driver gene fusions in breast cancer

After testing the performance of RWCfusion, we applied it to breast cancer to do case study. We used Tophat-Fusion to get candidate gene fusions from pair-end RNA-seq data of breast cancer samples. We combined the gene fusions of different breast cancer samples, discarding the fusion pairs appeared in normal sample. We totally got 52 non-redundant gene fusions from six breast cancer samples and only one gene fusion NOL8P1-NOL8, which was not in breast cancer samples, in normal sample. So we initially found 52 candidate gene fusions of breast cancer through Tophat-Fusion. As for high risk gene fusions, we got 59 of breast cancer from the total 483 high risk gene fusions.

We mapped the partner genes of the 52 gene fusions of breast cancer obtained from Tophat-Fusion into the gene interaction network. 31 of them were successfully mapped and considered as final candidate gene fusions of breast cancer. Then we used RWCFusion to score and rank these candidate gene fusions according to their global functional similarity with the high-risk breast cancer gene fusions in the weighted PPI network.

**Figure 3:**
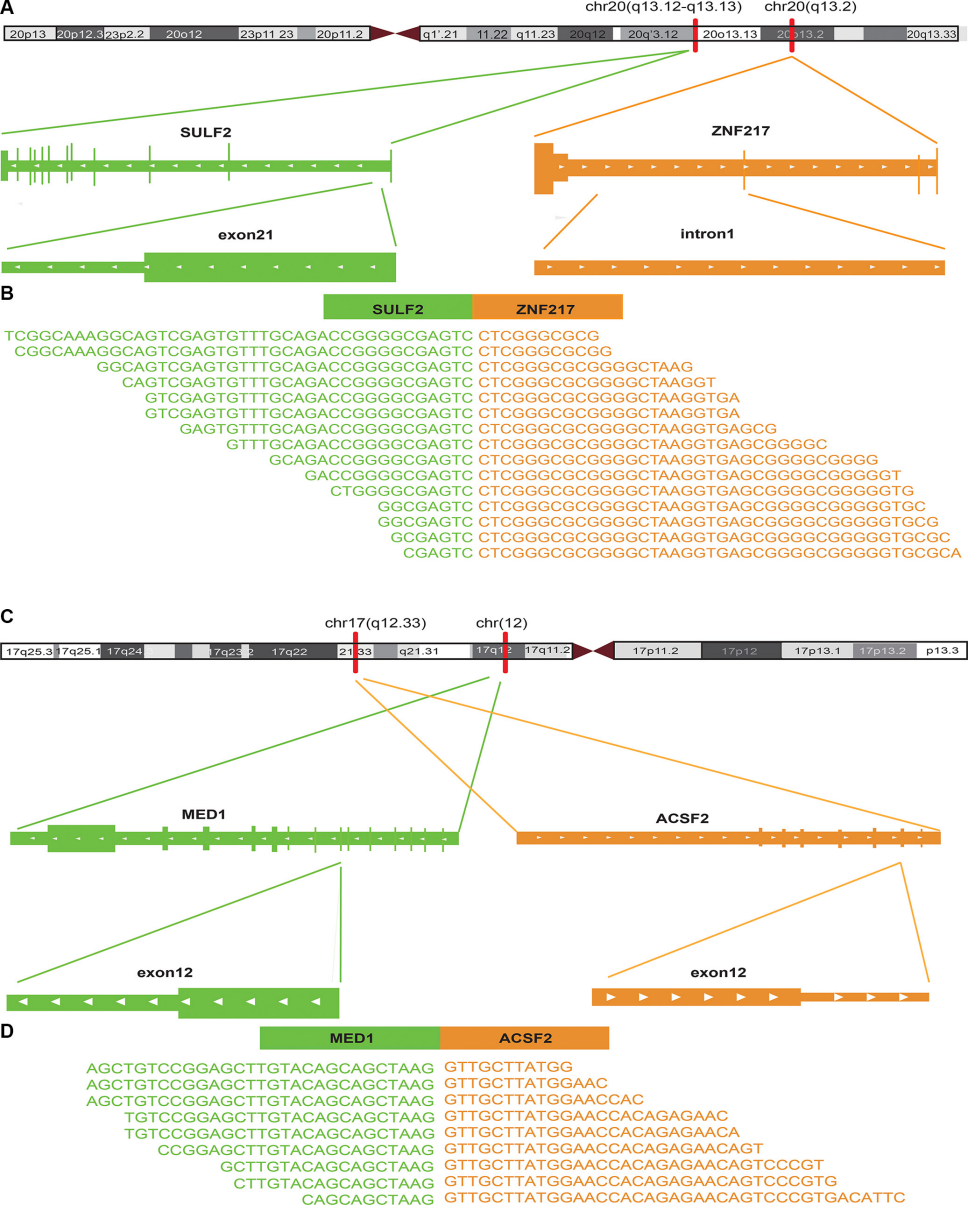
The fusion sites of the top 2 potential driver gene fusions of breast cancer identified by RWFusion (**A**, **C**) showed the chromosome location the fusion happened. (**B**, **D**) showed the reads that covered the breakpoints.

Using RWCFusion, we identified 13 previously known high-risk breast cancer gene fusions, ranking in the top 13 with high scores among the 31 mapped candidates (Table 3). For example, the fusion BCAS3-BCAS4 (breast carcinoma amplified sequence 3/4), whose partner gene BCAS3 and BCAS4 were both widely known as overexpression sequence and fusion in breast cancer [17], detected in MCF-7 cell line was nominated as top one fusion driver by RWCFusion. Notably, apart from the top 13, which have been proved to be high-risk breast cancer gene fusions according to ChiTaRS, we analyzed top 10 of the remaining candidate gene fusions of breast cancer whose biological roles had not been reported according to ChiTaRS, a database of fusion events in human cancers. To explore these 10 gene fusions, we searched literatures for each of them. And we found that 8 of them have potential possibility to be driver gene fusions of breast cancer (Table 4, Figure 3), the details are as following. In Table 4, the top 4 gene fusions each contains a partner gene which is also involved in a high-risk gene fusion of breast cancer, which may suggest that these 4 gene fusion may also play an important role in the occurrence and development of breast cancer. To examine the effects of a possible function of the partner genes of these 4 gene fusion in the breast cancer, we manually searched literatures and the result showed that: 1. SULF2 may act as a breast cancer suppressor, and knock-down of SULF2 in cell lines causes tumorigenic phenotypes, including increased proliferation, enhanced survival, and increased anchorage-independent growth [18]. 2. MED1 plays an important role in mediating resistance to the pure anti-estrogen fulvestrant both *in vitro* and *in vivo* and knockdown of MED1 potentiated tumor growth inhibition by fulvestrant [19]. 3. ACACA is a target gene of BRCA1, preventing its dephosphorylation through BRCA1 protein banding to it, while BRCA1 is widely known as a breast cancer susceptibility gene [20]. 4. STARD3 overexpression results in increased cholesterol biosynthesis and Src kinase activity in breast cancer cells and suggest that elevated StARD3 expression may contribute to breast cancer aggressiveness by increasing membrane cholesterol and enhancing oncogenic signaling [21]. Taken together, these top four gene fusions, containing one partner gene involved in the high-risk gene fusions of breast, have a partner gene playing as a suppressor or elevated role in the occurrence and development of breast cancer.

**Table 3:** The previously known high-risk gene fusions of breast cancer identified by RWCFusion

left gene	right gene	score
BCAS3	BCAS4	0.006677
NOTCH1	NUP214	0.006672
MED13	BCAS3	0.006652
CARM1	SMARCA4	0.006652
RPS6KB1	SNF8	0.006636
VMP1	RPS6KB1	0.006635
ARFGEF2	SULF2	0.006611
GLB1	CMTM7	0.006606
MED1	STXBP4	0.006604
VAPB	IKZF3	0.006588
PKIA	RARA	0.006582
MYO9B	RAB22A	0.006577
CYTH1	EIF3H	0.006574

**Table 4:** The top 10 of remaining gene fusions of breast cancer ranked by RWCFusion apart from the previously known high-risk gene fusions in Table 3

left gene	left chr	left coordinates	right gene	right chr	right coordinates	RWCFusion score
SULF2*	20q13.12-q13.13	46415148	ZNF217	02q13	52210294	0.000333
					52210645	
MED1*	17q12	37595417	ACSF2	17q21	48548388	0.00332
ACACA*	17q12	35479452	STAC2	17q12	37374425	0.00329
STARD3*	17q12	37793483	DOK5	20q13	53259996	0.00329
USP32#	17q23	58342772	PPM1D	17q23	58679978	0.0000642
					46371087	
THRA#	17q21	38243105	SKAP1	17q21	46371708	0.0000567
					46384692	
PPP1R12A	12q21	80211173	SEPT10	2q13	110343414	0.0000322
AHCTF1	1q44	247094879	NAAA	4q21	76846963	0.0000241
TOB1#	17q21	48943418	SYNRG	17q12	35880750	0.0000238
SUMF1#	3p26	4418013	LRRFIP2	3p22	37170639	0.0000161

* Partner genes that involved in a previously known high-risk gene fusion of breast cancer according to CHiTaRs at the same time.

# Partner genes that play important roles in the occurrence and development of breast cancer according to literatures evidence.

Apart from the top 4 gene fusions in Table 4, the other all gene fusions in this table contain no partner genes involved in any high-risk gene fusions of breast cancer. To investigate the potential function the remaining 6 gene fusions, we manually searched literatures for each of them. The results showed that 4 of them contained a partner gene that play an important role in the occurrence and development of breast cancer. 1. USP32 was found to be overexpressed more than twofold in 9 of 18 breast cancer cell lines compared to normal breast tissue, and upregulation of USP32 in mammary epithelial cells may be important in pathogenesis of breast cancer and/or serve as a useful biomarker in breast cancer cells [22]. 2. Few studies have addressed the question of the impact of THRA copy number variation in breast cancer, but it is reported to be amplified with HER2 in 50 to 80% of HER2-amplified breast cancers [23]. 3. TOB1 is regulated by EGF-Dependent HER2 and EGFR signaling and TOB1 protein expression and phosphorylation is associated with EGF-dependent erbB signaling and proliferation in breast cancer [24]. 4. SUMF1 is located in aUPD regions, which is a common and non-random molecular feature of breast cancer [25].

As Table 4 showed that the 8 gene fusions whose partner gene is associated with breast cancer are all involved in chromosome 17q. It has been reported that loss of heterozygosity on 17q region has been found in breast cancer [26]. This is consistent with the fact that 8 potential driver gene fusions of breast cancer we found are all involved in chromosome 17q. Taken together, we found 13 previously known high-risk gene fusions of breast cancer such as BCAS3-BCAS4, NOTCH- NUP214, MED13- BCAS3 and CARM-SMARCA4, and 8 potential drivers such as SULF2-ZNF217, MED1-ACSF2 (Table 3, Table 4).

## DISCUSSION

Gene fusions, one of human genomic aberrations, are believed to be causal factors of a variety of diseases. Identification of driver fusion genes associated specific cancer is still a challenge. It is believed that genes related to a specific or similar disease phenotype tend to be located in a specific neighborhood [12]. In this work, we proposed a network based method named RWCFusion to identify phenotype-specific cancer driver fusions. Our method assigns a score which reflects the global similarity of candidate gene fusion to known high risk fusions of a cancer to each candidate. In the beginning, the scores are 1 for seed high risk partner genes and 0 for candidate partners. They would be reassigned during the iteration in the process of RWCFusion. Two important elements determined the final score assigned to the candidate gene fusions: the weight involved distance to seed nodes and the number of seed nodes near the candidates. Scores of fusion pairs is the integrated results of their partner genes. A candidate gene fusion pair with a high score is close to the known high risk gene fusions of disease at a global scale. So it is more likely to be a driver gene fusion of tumor progression. The largest advantage of our method is that it can fully exploit interaction information of the network during the iteration at a global scale. This indicates RWCFusion can miner more information contained in the network than local network based method.

Many exploration had been made in the field of gene fusion research. For example, some previous studies often evaluated the oncogenic role of gene fusions by their recurrence, but limited success has been made [11]. Recent findings showed that many fusions important for development of cancer are non-recurrent, and some recurrent fusion genes founded from cancer cells also present in normal cells [27]. Apart from exploring the recurrence of gene fusion, there are also some other attempts have been made to characterize gene fusion. For example, fusion centrality method [13] similar to ours was proposed to distinguish ‘driver’ from ‘passenger’ gene fusions. We compared our method RWCFusion with fusion centrality, the results showed RWCFusion performed better than fusion centrality.

RWCFusion can be improved in the following directions. Firstly, it depends on the topology of the gene interaction network, so the low-quality and incompleteness of relation information may limit its performance. The performance could be further improved after more accurate and complete reconstructions of the network being made. Secondly, the quality and number of high-risk cancer gene fusions from the database might have influence on the performance. The continuing endeavor for accurate and quantified high-risk fusion information would further enhance our method. We hope that it will facilitate the research of mechanism of cancer development, potential prognostic and therapeutic targets in anti-cancer treatment.

## MATERIALS AND METHODS

### Gene interaction network

To get gene interaction network, we first downloaded the human protein interaction data (9606.protein.links.detailed.v9.0.txt.gz) from STRING (http://string-db.org/) database [14] and converted it into gene interaction network. STRING (The Search Tool for the Retrieval of Interacting Genes, v9.0) acting as a “one-stop shop”, provides integrated information of distinct types and sources of protein–protein association [14]. Several other resources that are currently being actively maintained are similar to STRING, such as: VisANT [28], GeneMANIA [29], N-Browse [30], I2D [31], APID [32] and ConsensusPathDB [33]. However, STRING is one of the very few sites to hold experimental, predicted and transferred interactions, together with interactions obtained through text mining. What's more, the links are weighted in the database, which will help to identify the most important nodes of interest in the network.

The PPI in STRING database are weighted with eight feature scores ranging from 0 to 1000, including neighborhood, fusion, co-occurence, co-expression, experimental database, text-mining and combined score which is the comprehensive score generated by STRING [14]. Here we choose “combined score” as the final weight, and the higher the score is, the closer the relationship of the two proteins is. After getting the weighted PPI network, we converted it into corresponding gene interaction network. Specially, two genes were linked if the proteins they encode interact with each other in STRING database. If two genes are corresponding to several different proteins respectively, the weight of this gene interaction pair is the mean value of the weights of their related protein interactions. And if two proteins are encoded by the same gene, it would be linked to itself after converting protein to gene. This kind of relationship would next be discarded in the gene interaction network. Finally the weight of edges in the network were normalized to 0–1 through dividing the weight in the protein interaction network by 1000.

### Obtain high-risk gene fusions of cancers

**Figure 4:**
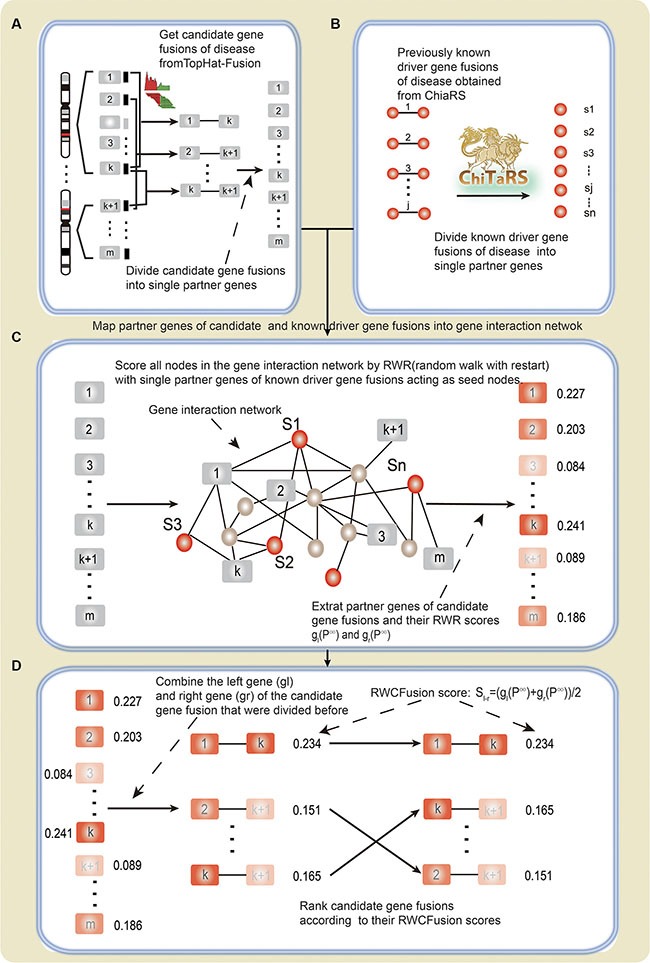
The flow diagram of RWCFusion (**A**) candidate gene fusions. (**B**) high-risk gene fusions. (**C**) and (**D**). the process of coring by RWCfusion. The nodes with rectangle shape represent partner genes of candidate gene fusions predicted by TopHat-Fusion, and the color of them reflect their scores by RWR: gray represent initial scores 0 and different level of red color reflects scores after RWR, the deeper the red color is, the higher RWR score it has. The nodes with circle shape represent partner genes of previously known high-risk gene fusions. The red color represent their initial score 1 before RWR. The straight line between two nodes (B, D) indicate that they are known or predicted to be fused.

We downloaded gene fusions of cancers from ChiTaRS database (http://chitars-old.bioinfo.cnio.es/), which contains human cancer breakpoints. The breakpoints in ChiTaRS were extracted from the TICdb [4], dbCrid [5], ChimerDB 2.0 [6] and Mitelman [1] databases. ChiTaRS database stores totally 1892 human cancer breakpoints, which were screened out manually through inspecting more than 7000 articles [7]. We obtained all human cancer breakpoints and their corresponding phenotypes from ChiTaRS database Figure 4B. Furthermore, we use OMIM database to normalize disease names and removed duplicated fusion genes within a certain phenotype, as different breakpoints may lie in same genes. Afterwards, we discarded those fusions with one or both of its partner genes not appearing in the gene interaction network. Finally, we screened out the phenotypes that had at least two gene fusions. (See Supplemental Information)

Given the fact that human cancer fusion events in ChiTaRS database were confirmed [7] as described before, we defined fusion pairs recorded in ChiTaRS database as “high-risk” human cancers gene fusions.

### RNA-Seq data

We analyzed the RNA-seq data from breast cancer, which contained seven samples related to 4 breast cancer and 1 normal cell lines. And the accession number of the breast cancer pair-end RNA-seq data is SRP003186 in SRA database. The samples in the data are one normal sample and 6 breast cancer sample related to 4 cell lines, including SK-BR-3, BT-474, MCF-7 and KPL-4. There were two biological replicates for BT-474 and SK-BR-3 each, of which the length of library fragments is 100 bp and 200 bp respectively.

### Obtain candidate gene fusions

Before identifying drivers from candidate gene fusions, the key step is actually to obtain candidate gene fusions. Tophat-Fusion is an algorithm developed to predict gene fusions by searching transcripts spanning gene fusion site and predict gene fusions by aligning pair-end RNA-seq reads without relying on existing annotation to genome [8]. Here we accessed Tophat-Fusion through Tophat-2.0.6, and the annotation file of the human genome is UCSC genome browser, hg19. We used TopHat-Fusion to obtain gene fusions from RNA-seq Figure 4A. Fusions that detected only in cancer samples compared to normal samples are defined as candidate fusions.

### The RWCfusion method

We developed RWCFusion method to identify cancer driver gene fusions in different cancers from candidates according to their global functional similarity with high-risk cancer gene fusions in the gene interaction network (Figure 4A–4D).

A gene fusion is hybridized by two previously separate genes, the effect on disease of a fusion can be inferred by its partners [11, 13]. First, we divided the high-risk and candidate driver gene fusion pairs of disease into single genes. If one or both partner genes in the of the high-risk and candidate cancer gene fusion pairs were not in the gene interaction network, the fusion pair would be discarded later on.

Second, we mapped single genes of divided high-risk and candidate disease driver gene fusions into the gene interaction network, and took partner genes of high-risk disease gene fusions as seed nodes and then employed a random walk with restart (RWR) [34] to score all the nodes in the network according to their global functional similarity with the seed nodes. After scoring all nodes in the network, we extracted the partner genes of divided candidate disease gene fusions and their scores in *p*^∞^. RWR simulates as an iterative walker transiting from current node to a randomly selected neighbor starting at a given seed node s, with an additional allow the restart of the walk in each step at node s with probability *β*. Formally, the random walk with restart is defined as:

$$p_{t+1}=(1-\beta )W^{T}p_{t}+\beta p_{0}$$ (1)

Where *W* represented the normalized adjacent matrix of the gene interaction network and *W_ij_* is the weight between gene *i* (*g_i_*) and gene *j* (*g_j_*). Here *p*_0_ is the initial probability vector and *p_t_* is a vector in which the *i– th* element holds the probability of random walker being at node *i* at step *t*. Here, the *i–th* element in *p*_0_ is 1 if *g_i_* is seed node and 0 if it is non-seed. Parameter *β* is the restart probability ranging from 0 to 1. At each step, the random walker can return to seed nodes with probability *β*. The probability will reach a steady state *p*_∞_ by performing the iteration until the variation between *p_t+1_* and *p_t_* (measured by L1 norm) is less than 10^−10^.

Third, we integrated the scores of the left partner genes (*g_l_*) and the right partner genes (*g_r_*) of the candidate gene fusions that were divided before to get the sores of the gene fusion. The scores of the candidate gene fusions *g_l_* – *g_r_* were defined as:

$$S_{l-r}=\frac{g_{l}(p^{\infty })+g_{r}(p^{\infty })}{2}$$ (2)

Where *S_l–r_* was the final score of the gene fusion between *g_l_* and *g_r_*. *g_l_* (*p*^∞^) is the final score of *g_l_* in RWR, whereas *g_r_* (*p*^∞^) is that of *g_r_*. Candidate gene fusions were ranked according to *S_l–r_*. The gene fusion with higher score would be more possible to be driver gene fusion of the disease.

### Performance measurement

To evaluate the performance of RWCFusion, we used leave-one-out cross-validation for every high-risk gene fusion. For every phenotype, each of the high risk gene fusion would be taken as held-out fusion in each test case. For each test case, the remaining high risk gene fusions were used as seed gene fusions. Then we generate virtual candidate fusion genes by randomly selecting gene pairs from the gene network. These randomly generated virtual fusion genes were considered as negative set of test, while the held out one was positive set of test in each test case. We calculated RWCFusion scores for every gene fusion in the test set in the each test case.

The receiver operator characteristic (ROC) curve could be plotted and the area under this curve (AUC) could be calculated according to above results. The ROC curve plots the true-positive rate (TPR) versus the false-positive rate (FPR). The held out one gene fusion in each test case was considered as positive test set, while the randomly selected fusions were considered as negative test set. Taking the RWCFusion scores of all candidates in each test case of a phenotype together, we could plot ROC curve and calculate AUC for every phenotype respectively. And by taking the RWCFusion scores of all candidates in each test case of all phenotypes together, we could calculate the overall performance of RWCFusion. As we all know, cancers are clinically classified into two classes namely haematological and solid. So we also evaluated the performance of our method for this two classes respectively in the same way.

### Fusion centrality

Fusion centrality is a network analysis method to prioritize gene fusions and the author believe that a fusion is more likely to be an oncogenic driver if its two partner genes act like hubs in a network [13]. A new node in a network, representing the two partner genes, inherits all the linkages of its partner genes. The centrality of the new node represents the importance of a fusion in a network. Centrality of a fusion is defined as its total linkages, namely, degree. We will compare our method RWCFusion with fusion centrality in this work.

## SUPPLEMENTARY MATERIALS TABLES


