# Supplementary material for: RWCFusion: identifying phenotype-specific cancer driver gene fusions based on fusion pair random walk scoring method
Source: Oncotarget. 2016 Aug 5;7(38):61054–68. doi: 10.18632/oncotarget.11064 (PMC5308635; doi:10.18632/oncotarget.11064)
Supplement: Supplementary file 1 [file oncotarget-07-61054-s001.pdf]

RWCFusion: identifying phenotype-specific cancer driver gene fusions based on fusion pair random walk scoring method

Supplementary Materials

High-risk gene fusions of cancers

We searched the OMIM database for the involved diseases of the 1885 human cancer breakpoints and discarded breakpoints whose related disease names were not recorded in OMIM database, remaining 1601 breakpoints corresponding to totally 862 gene fusions. Then we removed the involved partner genes which were not in the gene interaction network and 505 gene fusions were left. After screening out the phenotypes that had at least two gene fusions, 483 high risk gene fusions corresponding to 35 phenotypes were finally left.

Supplementary Table S1: The number of high-risk gene fusions identified by RWCFusion when the cutoff is 0.0002125

OMIM ID or Class	Disease Name	Class	Number of Known Disease Gene Fusions	Number of Identified high-risk Disease Gene Fusions
113970	BURKITT LYMPHOMA; BL	H	2	2
114480	BREAST CANCER	S	59	0
131440	MYELOPROLIFERATIVE DISORDER, CHRONIC, WITH EOSINOPHILIA	H	14	11
137800	GLIOMA SUSCEPTIBILITY 1; GLM1	S	3	0
144700	RENAL CELL CARCINOMA, NONPAPILLARY; RCC	S	3	1
150699	LEIOMYOMA, UTERINE; UL	S	3	3
151400	LEUKEMIA,CHRONIC LYMPHOCYTIC; CLL	H	2	1
155601	MELANOMA,CUTANEOUS MALIGNANT, SUSCEPTIBILITY TO, 2; CMM2	S	4	0
159595	MYELOPROLIFERATIVE SYNDROME, TRANSIENT	S	4	4
167000	OVARIAN CANCER	S	21	0
176807	PROSTATE CANCER	S	30	19
181030	SALIVARY GLAND ADENOMA, PLEOMORPHIC	S	8	5
188550	THYROID CARCINOMA, PAPILLARY	S	16	14
211980	LUNG CANCER	S	6	0
215300	CHONDROSARCOMA	S	4	3
236000	LYMPHOMA, HODGKIN	H	2	0
254700	MYELOPROLIFERATIVE DISEASE, AUTOSOMAL RECESSIVE	H	2	0
259500	OSTEOGENIC SARCOMA	S	2	0
268210	RHABDOMYOSARCOMA 1; RMS1	S	4	4
268220	RHABDOMYOSARCOMA 2;RMS2	S	5	5
300813	SARCOMA, SYNOVIAL	S	5	5
300854	RENAL CELL CARCINOMA, Xp11-ASSOCIATED; RCCX1	S	3	3
601626	LEUKEMIA, ACUTE MYELOID; AML	H	118	72
605027	LYMPHOMA, NON-HODGKIN, FAMILIAL	H	7	7
607685	HYPEREOSINOPHILIC SYNDROME, IDIOPATHIC; HES	H	7	7
607785	JUVENILE MYELOMONOCYTIC LEUKEMIA; JMML	H	6	5
608232	LEUKEMIA, CHRONIC MYELOID; CML	H	10	8
612160	HISTIOCYTOMA, ANGIOMATOID FIBROUS	S	2	2
612219	EWING SARCOMA; ES	S	8	8
612237	CHONDROSARCOMA, EXTRASKELETAL MYXOID	H	2	2
612376	ACUTE PROMYELOCYTIC LEUKEMIA; APL	H	14	13
613024	FOLLICULAR LYMPHOMA, SUSCEPTIBILITY TO, 1; FL1	H	10	9
613065	LEUKEMIA, ACUTE LYMPHOBLASTIC; ALL	H	83	47
613488	MYXOID LIPOSARCOMA	S	6	4
614286	MYELODYSPLASTIC SYNDROME; MDS	H	8	5
	H		287	189
	S		196	80
	Overall		483	269

H: Haematological, S:Solid.

Supplementary Table S2: The assessment of the robustness of RWCFusion when removing edges from the gene interaction network

OMIM ID	Disease name/class	Ratio of edges deleted from the network		
		10%	30%	50%
159595	MYELOPROLIFERATIVE SYNDROME,TRANSIENT	0.999	0.992	0.994
613024	FOLLICULAR LYMPHOMA, SUSCEPTIBILITY TO, 1; FL1	0.994	0.987	0.988
613065	LEUKEMIA, ACUTE LYMPHOBLASTIC; ALL	0.949	0.947	0.951
113970	BURKITT LYMPHOMA; BL	0.997	0.997	0.995
114480	BREAST CANCER	0.767	0.768	0.726
131440	MYELOPROLIFERATIVE DISORDER, CHRONIC, WITH EOSINOPHILIA	0.998	0.994	0.992
137800	GLIOMA SUSCEPTIBILITY 1; GLM1	0.752	0.751	0.773
144700	RENAL CELL CARCINOMA, NONPAPILLARY; RCC	0.987	0.971	0.979
150699	LEIOMYOMA, UTERINE; UL	1	0.998	0.999
151400	LEUKEMIA,CHRONIC LYMPHOCYTIC; CLL	0.992	0.975	0.937
155601	MELANOMA,CUTANEOUS MALIGNANT, SUSCEPTIBILITY TO, 2; CMM2	0.777	0.72	0.771
167000	OVARIAN CANCER	0.605	0.585	0.603
176807	PROSTATE CANCER	0.916	0.908	0.897
181030	SALIVARY GLAND ADENOMA, PLEOMORPHIC	0.988	0.977	0.975
188550	THYROID CARCINOMA, PAPILLARY	0.997	0.997	0.996
211980	LUNG CANCER	0.917	0.935	0.818
215300	CHONDROSARCOMA	0.963	0.973	0.979
236000	LYMPHOMA, HODGKIN	0.631	0.71	0.75
259500	OSTEOGENIC SARCOMA	0.679	0.76	0.742
268210	RHABDOMYOSARCOMA 1; RMS1	1	1	0.982
268220	RHABDOMYOSARCOMA 2;RMS2	0.998	1	0.988
300854	RENAL CELL CARCINOMA, Xp11-ASSOCIATED; RCCX1	0.996	0.999	1
601626	LEUKEMIA, ACUTE MYELOID; AML	0.981	0.981	0.978
605027	LYMPHOMA, NON-HODGKIN, FAMILIAL	1	0.999	1
607685	HYPEREOSINOPHILIC SYNDROME, IDIOPATHIC; HES	0.995	0.987	0.995
607785	JUVENILE MYELOMONOCYTIC LEUKEMIA; JMML	0.994	0.961	0.996
608232	LEUKEMIA, CHRONIC MYELOID; CML	0.977	0.976	0.957
612160	HISTIOCYTOMA, ANGIOMATOID FIBROUS	1	0.992	0.97
612219	EWING SARCOMA; ES	0.997	0.996	0.999
612237	CHONDROSARCOMA, EXTRASKELETAL MYXOID	1	1	0.985
612376	ACUTE PROMYELOCYTIC LEUKEMIA; APL	0.997	0.996	0.993
613488	MYXOID LIPOSARCOMA	0.939	0.937	0.929
614286	MYELODYSPLASTIC SYNDROME; MDS	0.98	0.984	0.986
254700	MYELOPROLIFERATIVE DISEASE, AUTOSOMAL RECESSIVE	0.755	0.876	0.783
300813	SARCOMA, SYNOVIAL	1	1	1
	Solid	0.866	0.863	0.847
	Haematological	0.968	0.967	0.966
	Overall	0.923	0.921	0.915

Supplementary Table S3: The assessment of the robustness of RWCFusion when changing the restart probability β

OMIM ID	Disease name/class	AUC value				
		β = 0.1	β = 0.3	β = 0.5	β = 0.7	β = 0.9
159595	MYELOPROLIFERATIVE SYNDROME,TRANSIENT	0.998	0.997	0.995	0.997	0.994
613024	FOLLICULAR LYMPHOMA, SUSCEPTIBILITY TO, 1; FL1	0.998	0.999	0.999	0.997	0.998
613065	LEUKEMIA, ACUTE LYMPHOBLASTIC; ALL	0.937	0.948	0.951	0.949	0.951
113970	BURKITT LYMPHOMA; BL	0.997	0.997	0.992	0.997	0.997
114480	BREAST CANCER	0.744	0.764	0.782	0.784	0.779
131440	MYELOPROLIFERATIVE DISORDER, CHRONIC, WITH EOSINOPHILIA	0.991	0.996	0.996	0.997	0.994
137800	GLIOMA SUSCEPTIBILITY 1; GLM1	0.734	0.744	0.765	0.759	0.795
144700	RENAL CELL CARCINOMA, NONPAPILLARY; RCC	0.924	0.976	0.976	0.978	0.981
150699	LEIOMYOMA, UTERINE; UL	1	0.999	1	0.996	0.996
151400	LEUKEMIA,CHRONIC LYMPHOCYTIC; CLL	1	0.987	0.99	0.995	0.982
155601	MELANOMA,CUTANEOUS MALIGNANT, SUSCEPTIBILITY TO, 2; CMM2	0.87	0.817	0.755	0.703	0.723
167000	OVARIAN CANCER	0.626	0.626	0.62	0.624	0.604
176807	PROSTATE CANCER	0.903	0.916	0.917	0.914	0.908
181030	SALIVARY GLAND ADENOMA, PLEOMORPHIC	0.967	0.991	0.992	0.993	0.992
188550	THYROID CARCINOMA, PAPILLARY	0.997	0.997	0.994	0.995	0.996
211980	LUNG CANCER	0.895	0.91	0.93	0.914	0.928
215300	CHONDROSARCOMA	0.984	0.978	0.97	0.964	0.965
236000	LYMPHOMA, HODGKIN	0.641	0.677	0.659	0.657	0.611
259500	OSTEOGENIC SARCOMA	0.75	0.672	0.72	0.73	0.71
268210	RHABDOMYOSARCOMA 1; RMS1	0.999	0.996	0.997	0.997	1
268220	RHABDOMYOSARCOMA 2;RMS2	0.998	1	1	1	0.999
300854	RENAL CELL CARCINOMA, Xp11-ASSOCIATED; RCCX1	1	0.999	1	1	0.997
601626	LEUKEMIA, ACUTE MYELOID; AML	0.965	0.976	0.979	0.98	0.981
605027	LYMPHOMA, NON-HODGKIN, FAMILIAL	1	1	1	0.999	1
607685	HYPEREOSINOPHILIC SYNDROME, IDIOPATHIC; HES	0.996	0.998	0.994	0.999	0.998
607785	JUVENILE MYELOMONOCYTIC LEUKEMIA; JMML	0.998	0.996	0.994	0.994	0.992
608232	LEUKEMIA, CHRONIC MYELOID; CML	0.979	0.978	0.974	0.971	0.971
612160	HISTIOCYTOMA, ANGIOMATOID FIBROUS	1	0.997	1	1	1
612219	EWING SARCOMA; ES	1	0.999	1	1	0.999
612237	CHONDROSARCOMA, EXTRASKELETAL MYXOID	1	1	1	1	1
612376	ACUTE PROMYELOCYTIC LEUKEMIA; APL	0.99	0.996	0.996	0.996	0.998
613488	MYXOID LIPOSARCOMA	0.947	0.953	0.943	0.936	0.932
614286	MYELODYSPLASTIC SYNDROME; MDS	0.938	0.968	0.979	0.979	0.981
254700	MYELOPROLIFERATIVE DISEASE, AUTOSOMAL RECESSIVE	0.937	0.869	0.818	0.735	0.775
300813	SARCOMA, SYNOVIAL	0.998	0.998	0.998	0.996	1
	Solid	0.852	0.865	0.867	0.867	0.861
	Haematological	0.961	0.968	0.971	0.968	0.965
	Overall	0.917	0.927	0.928	0.925	0.920
